# Identification of highly specific antibodies for Serine/threonine-protein kinase TBK1 for use in immunoblot, immunoprecipitation and immunofluorescence

**DOI:** 10.12688/f1000research.124632.1

**Published:** 2022-08-24

**Authors:** Walaa Alshafie, Maryam Fotouhi, Irina Shlaifer, Riham Ayoubi, Aled M. Edwards, Thomas M. Durcan, Peter S. McPherson, Carl Laflamme

**Affiliations:** 1 Department of Neurology and Neurosurgery, Structural Genomics Consortium, The Montreal Neurological Institute, McGill University, Montreal, Quebec, Canada; 2 The Neuro's Early Drug Discovery Unit (EDDU), Structural Genomics Consortium, McGill University, Montreal, Quebec, Canada; 3 Structural Genomics Consortium, University of Toronto, Toronto, Canada

**Keywords:** TBK1, Uniprot# Q9UHD2, antibody characterization, antibody validation, Western blot, immunoblot, immunoprecipitation, immunofluorescence

## Abstract

TBK1 is a serine-threonine protein kinase that has been linked to a number of diseases including amyotrophic lateral sclerosis and frontotemporal dementia. Reproducible research on TBK1 has been hampered by the lack of well characterized antibodies. In this study, we characterized 11 commercial antibodies for TBK1 for use in immunoblot, immunofluorescence and immunoprecipitation, using an isogeneic knock-out cell line as a control. We identify antibodies that appear specific for all three applications but invite the readers to interpret the present findings based on their own scientific expertise and use this report as a guide to select the most appropriate antibody for their specific needs.

## Introduction

The lack of robust characterization for research antibodies contributes to the reproducibility crisis. ^1^ Given that there are more than five million antibodies on the commercial market ( CiteAb.com), we hypothesize that with appropriate characterization criteria and testing, we should be able to identify high performing antibodies for many if not most proteins in the human genome. ^2^

TBK1 regulates autophagy through phosphorylation of Optineurin ^3^ and the C9ORF72/SMCR8 complex. ^4^ Of note, mutations in both Optineurin ^5^ and the C9ORF72/SMCR8 complex ^6^^,^^7^ cause monogenic forms of amyotrophic lateral sclerosis and frontotemporal dementia. Moreover, TBK1 also phosphorylates LC3C, GABARAP-L2 ^8^ and AKT1 ^9^ promoting autophagy.

The endogenous localization of TBK1 under the basal state and during autophagy remains to be determined. Moreover, TBK1 protein interactomes have been determined using overexpression systems, with the exception of one study. ^10^ TBK1 antibodies are key to address these unknowns.

To explore the availability of high-quality antibodies for human proteins, we devised an antibody characterization strategy in which we use wild-type (WT) and isogenic knockout (KO) control cells to perform head-to-head comparisons of all available commercial antibodies in immunoblot (Western blot), immunoprecipitation and immunofluorescence applications. ^11^ Here, we apply this approach to TBK1 and identify specific antibodies for all tested applications, enabling biochemical and cellular assessment of TBK1.

## Validation and discussion

To identify a cell line that expresses adequate levels of TBK1 protein to provide sufficient signal to noise, we examined the DepMap public proteomic database ( depmap.org, RRID:SCR_017655). U2OS was selected as the expression of TBK1 protein level is in the average range of cancer cells analyzed, ^12^ is easily amenable to CRISPR-Cas9 and is a rather flat cell line ideal for immunofluorescence studies. U2OS was modified with CRISPR/Cas9 to knockout the corresponding *TBK1* gene ( Table 1). ^13^

**Table 1.** Summary of the cell lines used.

Institution	RRID (Cellosaurus)	Cell line	genotype
Montreal Neurological Institute	CVCL_0042	U2OS	WT
Montreal Neurological Institute	CVCL_A6LQ	U2OS	*TBK1* KO

**Figure 1.**
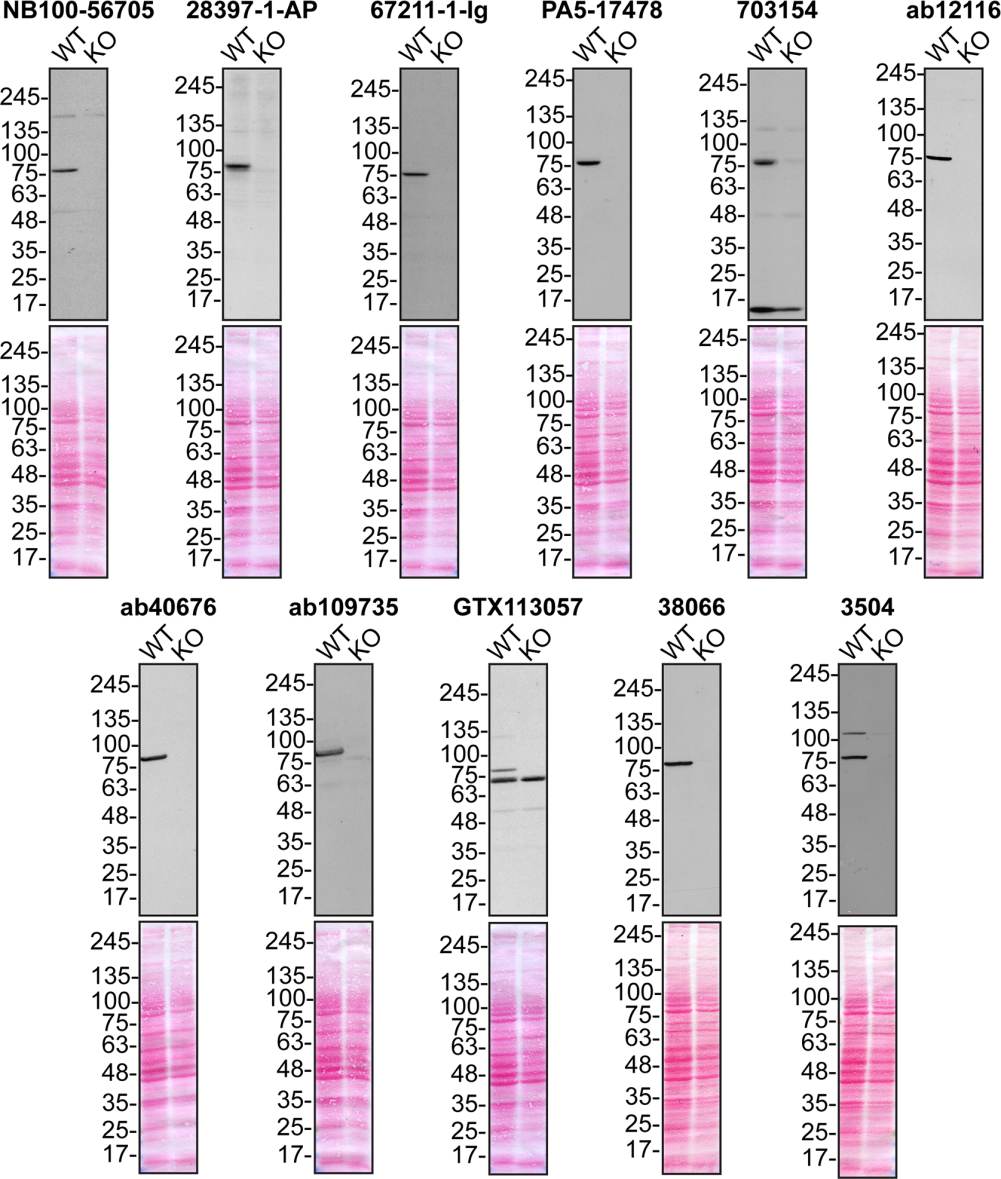
Serine/threonine-protein kinase TBK1 antibody screening by immunoblot. Lysates of U2OS (WT and *TBK1* KO) were prepared and 50 μg of protein were processed for immunoblot with the indicated TBK1 antibodies. The Ponceau stained transfers of each blot are presented to show equal loading of WT and KO lysates and protein transfer efficiency from the acrylamide gels to the nitrocellulose membrane. Antibody dilution used was 1/5000 for all tested antibodies. Predicted band size: ~83 kDa.

**Figure 2.**
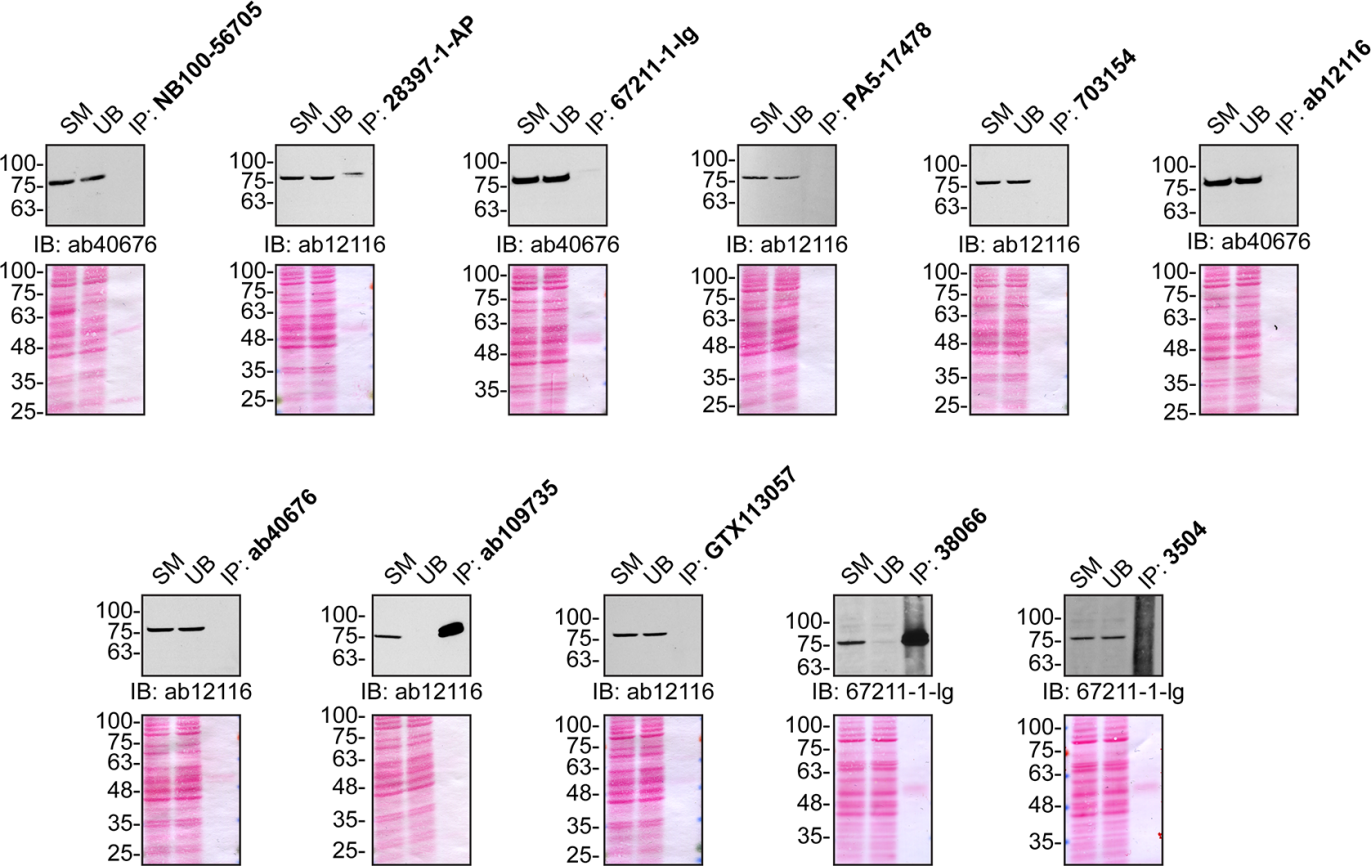
Serine/thronine-protein kinase TBK1 screening by immunoprecipitation. U2OS lysates were prepared and IP was performed using 1.0 μg of the indicated TBK1 antibodies pre-coupled to either protein G or protein A Sepharose beads. Samples were washed and processed for immunoblot with the indicated TBK1 antibody. For immunoblot, ab40676, ab12116 and 67211-1-Ig were used. The Ponceau stained transfers of each blot are shown for similar reasons as in Figure 1. SM=10% starting material; UB=10% unbound fraction; IP=immunoprecipitate.

**Figure 3.**
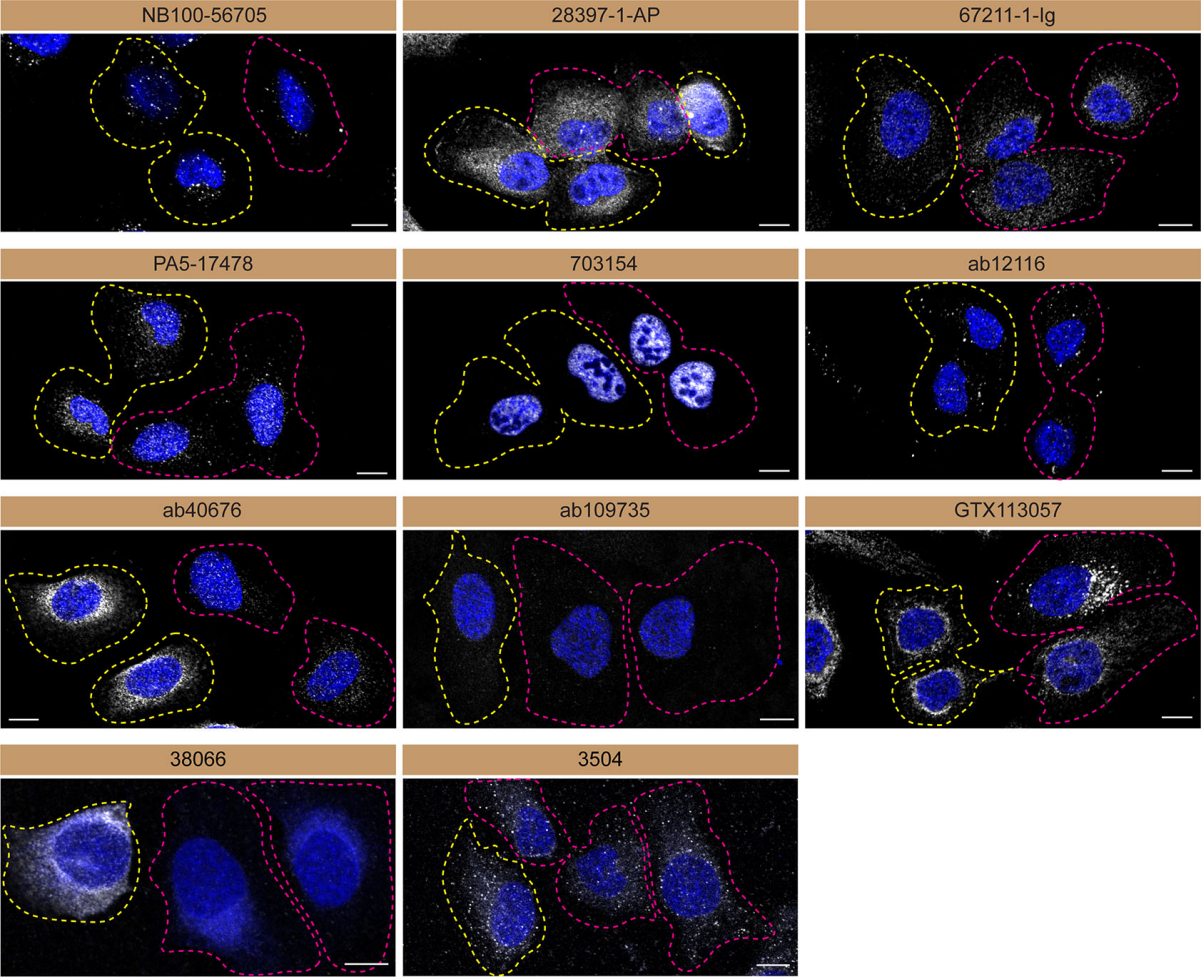
Serine/threonine-protein kinase TBK1 antibody screening by immunofluorescence. U2OS WT and *TBK1* KO cells were labelled with a green or a far-red fluorescent dye, respectively. WT and KO cells were mixed and plated to a 1:1 ratio on coverslips. Cells were stained with the indicated TBK1 antibodies and with the corresponding Alexa-fluor 555 coupled secondary antibody including DAPI. Acquisition of the blue (nucleus-DAPI), green (WT), red (antibody staining) and far-red (KO) channels was performed. Representative images of the merged blue and red (grayscale) channels are shown. WT and KO cells are outlined with yellow and magenta dashed line, respectively. Schematic representation of the mosaic strategy used is shown on the bottom-right panel. Antibody dilution used: NB100-56705 at 1/1000; 28397-1-AP at 1/500; 67211-1-Ig at 1/1000; PA5-17478 at 1/1000; 703154 at 1/500; ab12116 at 1/1000; ab40676 at 1/1500; ab109735 at 1/500; GTX113057 at 1/700, 38066 at 1/500, 3504 at 1/500. Bars = 10 μm.

Extracts from wild-type and *TBK1* KO cells were prepared and used to probe 11 commercial antibodies from 6 companies ( Table 2) by immunoblot (Western blot) and immunoprecipitation. The profile of each of the antibodies is shown in Figures 1, 2 and 3.

**Table 2.** Summary of the Serine/threonine-protein kinase TBK1antibodies tested.

Company	Catalog number	Lot number	RRID (Antibody Registry)	Clonality	Clone ID	Host	Concentration (μg/μl)	Vendors recommended applications
Bio-Techne	NB100-56705	B-1	AB_838420	monoclonal	108A429	mouse	1.00	Wb, IF
Proteintech	28397-1-AP	00076443	AB_2881132	polyclonal	-	rabbit	0.43	Wb
Proteintech	67211-1-Ig	10013180	AB_2882504	monoclonal	2D7B1	mouse	1.00	Wb, IF
Thermo Fisher Scientific	PA5-17478	VL3152289A	AB_10981817	polyclonal	-	rabbit	not provided	Wb, IF, IP
Thermo Fisher Scientific	703154	2274494	AB_2848223	recombinant-mono	JM42-11	rabbit	0.50	Wb, IF
Abcam	ab12116	GR3334526-1	AB_298856	monoclonal	108A429	mouse	1.00	Wb
Abcam	ab40676	GR3275777-2	AB_776632	recombinant-mono	EP611Y	rabbit	1.48	Wb, IF
Abcam	ab109735	GR3263881-3	AB_10863562	recombinant-mono	EPR2867(2)-19	rabbit	0.50	Wb, IP
GeneTex	GTX113057	43481	AB_11174793	polyclonal	-	rabbit	0.70	Wb, IF
Cell Signaling Technology	38066	1	AB_2827657	recombinant-mono	E8I3G	rabbit	not provided	Wb, IP, IF
Cell Signaling Technology	3504	-	AB_2255663	recombinant-mono	D1B4	rabbit	not provided	Wb, IP

Wb=Western blot; IF=immunofluorescence; IP=immunoprecipitation.

Antibodies were screened by immunofluorescence using a mosaic strategy. ^11^ WT cells were labelled with a green fluorescent dye, where as the KO cells were labelled with a far-red fluorescent dye. A third channel was used to image the primary antibodies. Plating WT and KO cells together and imaging both cell type in the same field of view reduces imaging and analysis biases.

In conclusion, we have screened TBK1 commercial antibodies by immunoblot, immunoprecipitation and immunofluorescence. The data provided can be used as a guide to purchase the most appropriate antibody for a researcher's needs.

## Methods

### Antibodies

All TBK1 antibodies are listed in Table 2. Peroxidase-conjugated goat anti-mouse and anti-rabbit antibodies are from Thermo Fisher Scientific (cat. number 65-6120 and 62-6520). Alexa-555-conjugated goat anti-mouse and anti-rabbit secondary antibodies are from Thermo Fisher Scientific (cat. number A21424 and A21429).

### CRISPR/Cas9 genome editing

Cell lines used are listed in Table 1. U2OS *TBK1* KO clone was generated using an open-access protocol ^13^ with an inducible Cas9 U2OS line. ^11^ Two guide RNAs (purchased at Synthego) were used to introduce a STOP codon in the *TBK1* gene (sequence guide 1: UUUGAACAUCCACUGGACGA, sequence guide 2: CAAAUUAUUUGCUAUUGAAG).

### Cell culture

Cells were cultured in DMEM high-glucose (GE Healthcare cat. number SH30081.01) containing 10% fetal bovine serum (Wisent, cat. number 080450), 2 mM L-glutamate (Wisent cat. number 609065, 100 IU penicillin and 100 μg/ml streptomycin (Wisent cat. number 450201).

### Antibody screening by immunoblot

Immunoblots were performed as described in our standard operating procedure. ^14^ Lysates were sonicated briefly and incubated 30 min on ice. Lysates were spun at ~110,000×g for 15 min at 4°C and equal protein aliquots of the supernatants were analyzed by SDS-PAGE and immunoblot. BLUelf prestained protein ladder from GeneDireX (cat. number PM008-0500) was used.

Immunoblots were performed with large 5-16% gradient polyacrylamide gels and transferred on nitrocellulose membranes. Proteins on the blots were visualized with Ponceau staining which is scanned at 300 dpi using a regular flatbed scanner to show together with individual immunoblot. Blots were blocked with 5% milk for 1 hr, and antibodies were incubated O/N at 4°C with 5% bovine serum albumin in TBS with 0.1% Tween 20 (TBST). Following three washes with TBST, the peroxidase conjugated secondary antibody was incubated at a dilution of ~0.2 μg/ml in TBST with 5% milk for 1 hr at room temperature followed by three washes with TBST. Membranes are incubated with ECL from Pierce (cat. number 32106) prior to detection with HyBlot CL autoradiography films from Denville (cat. number 1159T41).

### Antibody screening by immunoprecipitation

Immunoprecipitation was performed as described in our standard operating procedure. ^15^ Antibody-bead conjugates were prepared by adding 1.0 μg of antibody to 500 ul of PBS with 0.01% triton X-100 in a microcentrifuge tube, together with 30 μl of protein A- (for rabbit antibodies) or protein G- (for mouse antibodies) Sepharose beads. Tubes were rocked O/N at 4°C followed by several washes to remove unbound antibodies.

U2OS WT were collected in HEPES buffer (20 mM HEPES, 100 mM sodium chloride, 1 mM EDTA, 1% Triton X-100, pH 7.4) supplemented with protease inhibitor. Lysates are rocked 30 min at 4°C and spun at 110,000×g for 15 min at 4°C. One ml aliquots at 1.0 mg/ml of lysate were incubated with an antibody-bead conjugate for ~2 hrs at 4°C. Following centrifugation, the unbound fractions were collected, and beads were subsequently washed three times with 1.0 ml of HEPES lysis buffer and processed for SDS-PAGE and immunoblot on a 5-16% acrylamide gel.

### Antibody screening by immunofluorescence

Immunofluorescence was performed as described in our standard operating procedure. ^16^ U2OS WT and *TBK1* KO were labelled with a green and a deep red fluorescence dye, respectively. The fluorescent dyes used are from Thermo Fisher Scientific (cat. number C2925 and C34565). WT and KO cells were plated on glass coverslips as a mosaic and incubated for 24 hrs in a cell culture incubator. Cells were fixed in 4% PFA (in PBS) for 15 min at room temperature and then washed 3 times with PBS. Cells were permeabilized in PBS with 0,1% Triton X-100 for 10 min at room temperature and blocked with PBS with 5% BSA, 5% goat serum and 0.01% Triton X-100 for 30 min at room temperature. Cells were incubated with IF buffer (PBS, 5% BSA, 0,01% Triton X-100) containing the primary TBK1 antibodies O/N at 4°C. Cells were then washed 3 × 10 min with IF buffer and incubated with corresponding Alexa Fluor 555-conjugated secondary antibodies in IF buffer at a dilution of 1.0 μg/ml for 1 hr at room temperature with DAPI. Cells were washed 3 × 10 min with IF buffer and once with PBS. Coverslips were mounted on a microscopic slide using fluorescence mounting media (DAKO).

Imaging was performed using a Zeiss LSM 880 laser scanning confocal microscope equipped with a Plan-Apo 40× oil objective (NA = 1.40). Resulting images were cropped and adjusted for brightness and contrast using the Zen navigation software (Zeiss, Zen blue 3.4.91.00000). All cell images represent a single focal plane. Figures were assembled with Adobe Illustrator (version 26.3.1).

## Data availability

### Underlying data

Zenodo: Antibody Characterization Report for Serine/threonine-protein kinase TBK1, https://doi.org/10.5281/zenodo.6402968. ^17^

Zenodo: Dataset for the TBK1 antibody screening study, https://doi.org/10.5281/zenodo.6914815. ^18^

Data are available under the terms of the Creative Commons Attribution 4.0 International license (CC-BY 4.0).
