# Supplementary material for: Identification of highly specific antibodies for Serine/threonine-protein kinase TBK1 for use in immunoblot, immunoprecipitation and immunofluorescence
Source: F1000Res. 2022 Nov 24;11:977. Originally published 2022 Aug 24. [Version 2] doi: 10.12688/f1000research.124632.2 (PMC9647147; doi:10.12688/f1000research.124632.2)
Supplement: Peer review history

# Peer review: Reviewer response for version 2

**DOI:** 10.5256/f1000research.141225.r156538

**Published:** 2022-11-25

**Authors:** Nicolas Charlet

**Affiliations:** 1 Institut de Genetique et de Biologie Moleculaire et Cellulaire (IGBMC), INSERM , CNRS, Université of Strasbourg, Illkirch, France

The authors adequatey addressed my comments!

Are sufficient details of methods and materials provided to allow replication by others?

Yes

Is the rationale for creating the dataset(s) clearly described?

Yes

Are the datasets clearly presented in a useable and accessible format?

Yes

Are the protocols appropriate and is the work technically sound?

Yes

Reviewer Expertise:

Molecular Biology

I confirm that I have read this submission and believe that I have an appropriate level of expertise to confirm that it is of an acceptable scientific standard.

# Peer review: Reviewer response for version 1

**DOI:** 10.5256/f1000research.136844.r154820

**Published:** 2022-11-09

**Authors:** Simon L. Goodman

**Affiliations:** 1 The University of Turku, Turku, Finland

The researchers examine a set of 11 commercially available antibodies marketed as recognizing TBK1, a protein linked to several human neurological conditions, for reactivity in Western blot, immunoprecipitation, and fixed cell Immunofluorescence. 

 The specificity of the antibodies in the various techniques is shown beautifully, but not commented upon, which I find extremely strange. The YCharOs team follow a publication strategy of letting the results "speak for themselves".

 The data is clearly presented in a standard format used by the YCharOs consortium, and detailed protocols are available online. I dislike the use of truncated gels in the IP data set (Fig2). It would improve the credibility if the whole IPs were shown, maybe as supplementary data?

 Especially, the use of Table#2 including batch and RRID identifiers is an excellent example which all should follow. Would it be possible to have links in print for the RRIDs?

 I was rather puzzled by the choice of working concentrations, for the various techniques, which is not explained anywhere. Were all the reagents pre-titred in the techniques? Please, could the authors clarify a little?

 The appalling situation with commercial antibodies being inadequately validated is now quite widely known, so YCharOs efforts are laudable, and to be encouraged. For the naive reader, the team at YCharos have an admirable mission to correctly and independently validate commercial tool antibodies. Their model involves external funding from antibody suppliers and the Canadian government.

 This may be the source of my only criticism of the presentation method: it is a little odd, because the authors do not follow scientific traditions of discussing their data. One speculates that antibody providers, or managers within providers, of less-than-perfect antibodies might decide that negative commentary on their products was not worth the funding? But this does reduce the usability and accessibility of the data set. Can't the authors add a table summarizing the data sets (+ / - format stating apparent antibody specificity in each technology would do). Even without further detailed comment. I am perfectly well aware of why the authors have not chosen to do this; however, it is a little sad.

 Regardless: as a reviewer, I see that all the reagents WB well, but only 2-3 IP a band of an appropriate MW.  And only 2 are some way specific in pfa-IF.  Only one antibody, clone E813G, appears specific in all techniques.

 This shows the power of the YCharOs approach, and the importance of their validation work - which should of course be published.  This article could save the average researcher who need to find a specific TBK1 antibody for IF many thousands of dollars, and several weeks of frustrated effort, for example.

 Some minor points about the antibody table #2:

 108A429: this antibody has some 10 suppliers (CiteAb), but BioTechne is not one of them. Please clarify.

 2D7B1 = clone name also used by the supplier for another target specificity (TFF1).

 JM42-11 = anti- TFF1 from thermo fisher (and anti-TIA-1 from other companies) clone identifier and catalog number don't align. Catalog describes clone 12H60L39.

 Methods:

 IPs lysates: 110000g? Typo? g max? If not, please mention centrifuge used. "Several washes". Please specify wash buffer.

Are sufficient details of methods and materials provided to allow replication by others?

Yes

Is the rationale for creating the dataset(s) clearly described?

Yes

Are the datasets clearly presented in a useable and accessible format?

Partly

Are the protocols appropriate and is the work technically sound?

Yes

Reviewer Expertise:

Antibody validation, IHC, integrins,

I confirm that I have read this submission and believe that I have an appropriate level of expertise to confirm that it is of an acceptable scientific standard.

# Response

**Published:** 2022-11-16

**Authors:** Carl Laflamme

**Affiliations:** McGill University, Canada

We thank Simon Goodman (reviewer #2) for his review of our manuscript, and for summarizing the YCharOS effort.

 Dr. Goodman states “ *The specificity of the antibodies in the various techniques is shown beautifully, but not commented upon, which I find extremely strange. The YCharOs team follow a publication strategy of letting the results "speak for themselves*".

 As discusses in respond to Dr. Chartrand, we believe that scientists interested in reading our reports have the expertise to interpret the antibody characterization data.

 Dr. Goodman mentions: “ *I dislike the use of truncated gels in the IP data set (Fig2). It would improve the credibility if the whole IPs were shown, maybe as supplementary data?”*.

 We presented truncated gels in Figure 2 for space considerations. The complete lanes are shown in a collection of all raw data available to the public through the Zenodo open science repository. This link is also indicated in the article in the "Data Availability" section. This article was written under the "Data Notes" specification, and supplementary materials are not accepted. The posting of the supplementary materials on Zenodo was made in accordance with F1000's open data policy.

 Dr. Goodman states, “ *Especially, the use of Table#2 including batch and RRID identifiers is an excellent example which all should follow. Would it be possible to have links in print for the RRIDs?*“

 We thank Dr Goodman for his supportive comment. We have followed his recommendation and added the corresponding RRID link for each antibody to Table 2. Moreover, we confirmed that Novus (Bio-Techne) sells clone # 108A429 (cat# NB100-56705). We confirmed clone # 2D7B1 for Proteintech 67211-1-Ig. The clone number for Thermo 703154 is indeed 12H60L39 and not JM42-11 as originally indicated. This mistake was corrected in the revised version of the manuscript.

 Dr. Goodman mentioned to be “ *puzzled by the choice of working concentration*”.

 We agree and have clarified how we selected antibody concentration. For WB, we follow the antibody manufacturer’s recommendations. Most TBK1 antibodies are recommended at 1/500 to 1/2000. For six TBK1 antibodies, the signal-to-noise ratio was satisfactory following manufacturers’ recommendations. However, the signal was too strong for 28397-1-AP, PA5-17478, 703154, ab40676 and ab109735 which we titrated at 1/10000. The figure legend has been updated in the revised version of the manuscript. For IF, we tested a dilution of 1.0 µg/ml for all antibodies. At this concentration, the signal from each antibody was in the range of detection of the microscope used.

 Dr. Goodman mentioned that “ *it is a little odd, because the authors do not follow scientific traditions of discussing their data*”.

 In the F1000 Data Note format, discussion is not required, and we think it is not relevant for this presentation.

 Dr Goodman suggested “ *can't the authors add a table summarizing the data sets (+ / - format stating apparent antibody specificity in each technology would do*”).

 We do not score/recommend antibodies because we tested the antibodies under one set of conditions, and the scoring/recommendation would be valid only under this precise experimental setup and in the cell line used. That said, YCharOS reports serve as an invaluable guide pointing scientists to appropriate antibodies for their experimental needs. 

 Dr. Goodman speculates that “ *antibody providers, or managers within providers, of less-than-perfect antibodies might decide that negative commentary on their products was not worth the funding?”* 

 We can reassure Dr. Goodman and all readers and users of the YCharOS data that participating companies do not influence data presentation beyond informed scientific feedback. Every antibody tested is presented in the report, we omit no antibodies and never have. Prior to their release on Zenodo, each antibody characterization report is shared for technical review by a group of scientific advisors from academia and from participant antibody providers. Once the technical aspects have been reviewed, the reports, without edits, are uploaded without restriction on Zenodo. To date, antibody providers have actively removed sub-performing antibodies or modified recommendations after assessing our data. One TBK1 antibody has been already removed from the market, and recommendation has been modified for two TBK1 antibodies.

# Peer review: Reviewer response for version 1

**DOI:** 10.5256/f1000research.136844.r148432

**Published:** 2022-09-06

**Authors:** Nicolas Charlet

**Affiliations:** 1 Institut de Genetique et de Biologie Moleculaire et Cellulaire (IGBMC), INSERM , CNRS, Université of Strasbourg, Illkirch, France

In this work, Laflamme and collaborators investigate the quality and specificity of various commercial antibodies directed against the TBK1 protein, an important kinase regulating immunity and which loss- or gain-of-function mutations cause Amyotrophic Lateral Sclerosis or Normal-Tension Glaucoma, respectively.

 Importantly, this work revealed that while most of the tested antibodies are suitable for classical immunoblot, only two of them are usable for immunoprecipitation. Similarly, only two, maybe three, TBK1-antibodies are specific in a well-controlled immunofluorescence assay. This is an extremely important result as the use of an incorrect antibody will undoubtedly result in the wrong interpretation of TBK1 localization by immunofluorescence and/or identification of erroneous interactants in immunoprecipitation followed by mass spectrometry analyzes.

 Overall, the text is clear and well written, experiments are well presented and technically extremely well performed and controlled, notably by the use of cells knockout for TBK1 expression, which is classical in immunoblot assays, but when mixed with WT cells is extremely well thought to test antibodies specificity in immunofluorescence. My only recommendation would be to **include a table summarizing what antibody is best recommended for what usage**, as the current “Invitation to the readers to interpret authors finding” will be a source of misinterpretation, especially in immunoprecipitation and immunofluorescence assays.

Are sufficient details of methods and materials provided to allow replication by others?

Yes

Is the rationale for creating the dataset(s) clearly described?

Yes

Are the datasets clearly presented in a useable and accessible format?

Yes

Are the protocols appropriate and is the work technically sound?

Yes

Reviewer Expertise:

Molecular Biology

I confirm that I have read this submission and believe that I have an appropriate level of expertise to confirm that it is of an acceptable scientific standard.

# Response

**Published:** 2022-11-16

**Authors:** Carl Laflamme

**Affiliations:** McGill University, Canada

We thank Nicolas Charlet (reviewer #1) for review of our manuscript. Dr. Charlet suggests we “ *include a table summarizing what antibody is best recommended for what usage*” to avoid misinterpretation of our antibody characterization data.

 We have presented the YCharOS initiative on several occasions and have found that for the most part, scientists interested in our reports have the expertise to interpret the antibody characterization data. Moreover, we do not score/recommend antibodies because we tested the antibodies under one set of conditions, and the scoring/recommendation would be valid only under this precise experimental setup and in the cell line used. That said, YCharOS reports serve as an invaluable guide pointing scientists to appropriate antibodies for their experimental needs.
